# Predicting clinical pregnancy using clinical features and machine learning algorithms in in vitro fertilization

**DOI:** 10.1371/journal.pone.0267554

**Published:** 2022-06-08

**Authors:** Cheng-Wei Wang, Chao-Yang Kuo, Chi-Huang Chen, Yu-Hui Hsieh, Emily Chia-Yu Su

**Affiliations:** 1 Division of Reproduction Medicine, Department of Obstetrics and Gynecology, Taipei Medical University Hospital, Taipei, Taiwan; 2 Graduate Institute of Biomedical Informatics, College of Medical Science and Technology, Taipei Medical University, Taipei, Taiwan; 3 Smart Healthcare Interdisciplinary College, National Taipei University of Nursing and Health Sciences, Taipei, Taiwan; 4 Department of Obstetrics and Gynecology, School of Medicine, College of Medicine, Taipei Medical University, Taipei, Taiwan; 5 School of Nutrition and Health Sciences, College of Nutrition, Taipei Medical University, Taipei, Taiwan; 6 Clinical Big Data Research Center, Taipei Medical University Hospital, Taipei, Taiwan; Fondazione IRCCS Ca’ Granda Ospedale Maggiore Policlinico, ITALY

## Abstract

**Introduction:**

Assisted reproductive technology has been proposed for women with infertility. Moreover, *in vitro* fertilization (IVF) cycles are increasing. Factors contributing to successful pregnancy have been widely explored. In this study, we used machine learning algorithms to construct prediction models for clinical pregnancies in IVF.

**Materials and methods:**

A total of 24,730 patients entered IVF and intracytoplasmic sperm injection cycles with clinical pregnancy outcomes at Taipei Medical University Hospital. Data used included patient characteristics and treatment. We used machine learning methods to develop prediction models for clinical pregnancy and explored how each variable affects the outcome of interest using partial dependence plots.

**Results:**

Experimental results showed that the random forest algorithm outperforms logistic regression in terms of areas under the receiver operating characteristics curve. The ovarian stimulation protocol is the most important factor affecting pregnancy outcomes. Long and ultra-long protocols have shown positive effects on clinical pregnancy among all protocols. Furthermore, total frozen and transferred embryos are positive for a clinical pregnancy, but female age and duration of infertility have negative effects on clinical pregnancy.

**Conclusion:**

Our findings show the importance of variables and propensity of each variable by random forest algorithm for clinical pregnancy in the assisted reproductive technology cycle. This study provides a ranking of variables affecting clinical pregnancy and explores the effects of each treatment on successful pregnancy. Our study has the potential to help clinicians evaluate the success of IVF in patients.

## Introduction

### Background

Infertility is a global health issue that affects individuals, families, and society. The prevalence of infertility has increased in recent years. In the United States, the prevalence of infertility is approximately 8.8% (National Center for Health Statistics, 2020). In the United Kingdom, the prevalence of infertility was 12.5% in women and 10.1% in men [1]. In 2010, in 190 countries and territories worldwide, women aged 20–44 faced the possibility of pregnancy, and 1.9% were unable to experience a live birth [2]. In Taiwan, the female fertility rate decreased from 7.04 children per woman in 1951 to 1.165 in 2014. As a result, Taiwan has one of the lowest fertility rates worldwide [3]. Therefore, infertility has become a major healthcare issue in Taiwan.

Since the advent of assisted reproductive technologies (ARTs) in 1984, the use of *in vitro* fertilization (IVF) cycles has increased worldwide. Clinical pregnancy is defined as the pregnancy that lasted 6 weeks (or 42 days) after the onset of the last menstrual period and confirmed by human chorionic gonadotropin (hCG) assay [4]. Achieving high clinical pregnancy rates has been a major goal for both physicians and patients. Pregnancy rates after IVF treatment are approximately 30–70%, depending on the age of the female patient and the different types of interventions employed. Factors that contribute to successful pregnancies have been widely explored. Factors that affect the success of ARTs include age, body mass index (BMI), uterine and ovarian factors, the type of stimulation protocol used, the stimulation dose, and the use of fresh or frozen protocols. ART lies in the interaction of these variables to achieve higher numbers of oocytes and higher pregnancy rates. There are no fixed protocols for individual patient conditions.

While reducing or removing adverse factors for pregnancy, prediction models based on existing large-scale databases have been established to boost our understanding of IVF procedures and improve pregnancy rates [5]. The development of prediction models can help physicians provide personalized treatment for each infertility couple. Artificial intelligence (AI) is a technology suitable for application in ART fields for several reasons. ARTs require highly individualized protocols. Parameters affecting the success of ARTs may be discovered during the process of AI calculations. Moreover, by predicting the best pregnancy rates, individualized, tailored protocols can be used for infertile couples. This study aimed to utilize machine-learning algorithms to predict clinical pregnancy rates.

### Literature review

Machine learning is a computational method that focuses on how computers learn from data. It intersects with statistics, with the goal of discerning relationships from data and computer science, and emphasizes efficient computing algorithms [6]. Algorithms use AI and statistics to find patterns in a dataset. Data patterns are then used to make predictions [7]. Several previous studies proposed predicting pregnancy using IVF based on traditional statistical and machine-learning methods.

Logistic regression is a popular statistical method that develops prediction models. To construct a prediction model based on logistic regression, Ottosen *et al*. used 1,675 records containing IVF and intracytoplasmic sperm injection (ICSI) treatment cycles. Their variables included embryo quality, patient age, duration of infertility (months), BMI, basal follicle-stimulating hormone (FSH), treatment type, indication for treatment, number of oocytes retrieved, fertilization rate, and scores of the best and second-best embryos. Embryo quality, patient age, and basal FSH levels showed statistically significant effects on pregnancy. A receiver operating characteristics (ROC) curve was incorporated to evaluate the predictive performance, and the area under the ROC curve (AUC) values were 0.64 and 0.68 for singleton and twin pregnancy models, respectively [8]. Hansen *et al*. used a logistic regression to analyze conception, clinical pregnancies, and live births in a dataset that included 19 medical and socioeconomic variables of 900 couples with unexplained infertility. The AUC values of the prediction models for conception, clinical pregnancies, and live births were 0.66, 0.64, and 0.65, respectively [9]. Meijerink *et al*. developed a prediction model based on multivariate logistic regression using a dataset containing 289 couples with 553 testicular sperm extraction (TESE)-ICSI cycles. The dataset included types of infertility, duration of infertility (months), female age, parity, average menstrual cycle length (days), uterine abnormalities, antral follicle count before stimulation, alcohol use and smoking status for men and women, BMI at baseline for men and women, male age, male luteinizing hormone (LH), male inhibin levels, male FSH, total testicular volume, and a suspected primary diagnosis of azoospermia before sperm retrieval. The AUCs of the logistic regression prediction model were 0.62 and 0.67 for the validation data [10].

Compared to logistic regressions, machine learning algorithms are more sensitive and more specific screening techniques, for which the assumptions and restrictions of traditional regressions are relaxed. Machine learning algorithms have become increasingly common for learning from data to develop more reliable predictions [11]. In reproductive science, machine learning algorithms have also been incorporated in several studies. Blank *et al*. used a dataset collected by the Department of Reproductive Medicine, Ghent University Hospital, Belgium, containing 1,052 patients who underwent single-embryo transfer (SET) using fresh day-5 blastocysts. The dataset contained 32 variables, including continuous variables (male and female age and anti-Müllerian hormone [AMH]), categorical variables (stimulation protocols), and discrete variables (number of oocytes). To predict implantation after blastocyst transfer in IVF, a random forest algorithm showed better predictive performance than a logistic regression in terms of the AUC (0.74 for the random forest and 0.66 for the logistic regression) [12]. Qiu *et al*. applied variables, including age, AMH, duration of infertility, BMI, previous live births, previous miscarriages, previous abortions, and type of infertility (classified into tubal, anovulatory, male factor, and unexplained factors) with 7,188 records of women who were undergoing their first IVF treatment. To compare predictive performances based on the AUC, machine learning algorithms, including support vector machines, random forest, and extreme gradient boosting (XGBoost), outperformed traditional logistic regression in personalized predictions of live births prior to the first IVF treatment. Moreover, XGBoost and random forest algorithms achieved higher AUCs of approximately 0.73 compared to the other algorithms [13].

In addition to models for predicting pregnancy in ARTs, machine learning algorithms have also been incorporated to evaluate fetal health status. Akbulut *et al*. proposed an e-Health application based on machine learning algorithms to predict fetal anomaly status by referring to maternal and clinical data. Several binary prediction models were trained with a clinical dataset consisting of 96 pregnant women, and the highest accuracy achieved was 89.5% in a test set with a random forest model. In addition, the proposed model was applied to a real-life test of 16 users and obtained an accuracy of 87.5% [14]. This study aimed to incorporate machine learning algorithms to analyze independent variables influencing clinical pregnancy outcomes using ART, which may also provide useful information for clinicians and infertile couples.

## Materials and methods

### Dataset

Taipei Medical University Hospital (TMUH) is one of the leading hospitals in reproductive medicine in Taiwan, with more than 2,000 ART cycles conducted annually. Every IVF cycle was registered in a national health bureaucracy database. More than 100,000 IVF cycles were registered in the database. This study was approved by the Institutional Review Board (IRB) of TMUH (TMU-Joint Institutional Review Board N201908012). The IRB waived the requirement to obtain informed consent. In total, 24,730 patients underwent IVF/ICSI cycles with clinical pregnancy outcomes in the original data from the Health Promotion Administration, Ministry of Health, and Welfare. We used deidentified personal data and performed the study in accordance with the Declaration of Helsinki. As shown in Table 1, the clinical data of women aged 21–55 years who underwent IVF/ICSI cycles at TMUH were analyzed. Patients who underwent embryo transfer with both fresh and freeze-thawed embryos simultaneously in the same cycle of IVF treatment were excluded because the embryo origin could not be defined when assessing pregnancy outcomes. Moreover, we collect 3,352 new samples from 2020–2021 as an independent test set for external validation of our proposed model.

**Table 1:** Characteristics of the analytical variables.

Variable	Definition or Range
Male age (years)	23 to 78
Female age (years)	21 to 55
Duration of infertility (years)	1 to 14
Number of IVF cycles performed	0 to 16
Number of oocytes retrieved	0 to 52
The Number of Embryos Transferred	1 to 4
The Total Number of Frozen Embryos	0 to 36
Cause of infertility	Tubal factor, ovary factor, endometriosis, uterine factor (myoma adenomyosis, uterine synechia), others (other female factors), male factor, either two factors, unexplained
Fertilization method	IVF/ET (in vitro fertilization/embryo transfer), ZIFT/TET (zygote intrafallopian transfer/tubal embryo transfer)
Micromanipulation technique	ICSI (intracytoplasmic sperm injection), assisted hatching, ICSI and assisted hatching, PGT (preimplantation genetic testing), no use, others
Source of sperm and oocytes	Without donation, oocyte donation, sperm donation
The use of fresh/freeze-thaw	Fresh, freeze-thaw
Ovarian hyperstimulation syndrome	None, mild, moderate, severe
Ovarian stimulation protocol	Natural cycle (including frozen-embryo transfer cycle), oral stimulation drug, short protocol, long protocol, ultra-long protocol, antagonist protocol, others

### Imputation and data partition

In total, 7,362 records of missing values in ovarian stimulation protocols were imputed by the missForest function in the random forest algorithm. This random forest-based function can impute missing values and perform better compared to other methods of imputation, especially in datasets that consist of different types of variables and complex interactions or non-linear relationships [15]. The imputation of missing values plays an important role in the data analysis. Moreover, we used the functional strata of package sampling for random sampling to divide the original dataset into 50% as the training dataset for model development and 50% as the test dataset for model validation.

### Descriptive statistical analysis

Statistical analyses were conducted using the RStudio (2009–2018 RStudio) software version 1.1.463 and SAS version 9.4 (SAS Institute, Cary, NC, USA). Means and standard deviations (SDs) were calculated for continuous variables, and frequencies and percentages were computed for categorical variables. Baseline characteristics between participants with and without a clinical pregnancy were compared using a chi-squared test for categorical variables and Student’s *t*-test for continuous variables.

### Machine learning algorithms

To predict pregnancy outcomes after IVF, we compared the performances of prediction models built using logistic regression or random forest algorithms. Previous studies used logistic regressions to build prediction models and observed their results using odds ratios (ORs). We incorporated the random forest algorithm to develop predictive models because advantages of the random forest include that it converges due to the law of large numbers and shows no overfitting without pruning in its predictions [16]. This study aimed to accurately predict clinical pregnancies and to depict the partial effect of each variable on the outcome.

The random forest method is a combination of tree predictors, in which all trees are independently built by random vector sampling and have the same distribution in the forest. The generation error converges to a limit as trees in forests become larger. The classifier error in a forest depends on the strength and correlation between the trees [16]. Breiman proposed the random forest model in 2001, which can be created using the randomForest package, and is also available in the R environment [17]. The random forest model was constructed using the randomForest package [17]. We built the model using 1,000 trees, and three variables were sampled randomly in each tree. The importance of the variables in the model was measured by the mean decrease accuracy (MDA), which was calculated by how much accuracy was reduced when each variable was left out.

To depict the influence of different variables, the random forest model can generate the importance of variables by observing the out-of-bag (OOB) error of a specific variable, as the other predictors remain stable [17]. The importance of a variable depicts the rank of that variable in influencing pregnancy predictions when building a prediction model. After comparing the importance of variables, we analyzed how the variables affected the probability of a clinical pregnancy. The use of partial dependence plots is a good way to provide insights into each machine learning model, as they depict how each variable influences the prediction when all other variables are simultaneously averaged [18]. partialPlot is a function in the randomForest package, which depicts the marginal effects of variables on the class probability for classification. Plots show the relative logit distributions of class probabilities from the model. Positive values on the *y*-axis indicate that the values of the independent variables are more likely to be a positive class. In contrast, negative values are less likely to be a positive class. Zero indicates the absence of an average influence on the class probability.

### Hyperparameter tuning

One of the most important variables in the random forest algorithm is the *mtry* parameter, which is defined as the number of predictors sampled for splitting at each node. In our experiments, two approaches were incorporated for hyperparameter tuning of the *mtry* variable. The function “tuneRF” of the package “randomForest” and function “train” of package “caret” are two methods in tuning hyperparameter “mtry” for random forest algorithm. We used “tuneRF” to find the “mtry” with the minimum OOB error for 10 times and applied “train” to find the highest AUC with ten-fold cross-validations.

### Evaluation measures

Four measures, including accuracy, sensitivity, specificity, and AUC, were used to evaluate the predictive model. Accuracy, sensitivity, and specificity were generated from a function confusion matrix, and the AUC was generated from the ROCR package in the R environment. The accuracy, sensitivity, and specificity of the clinical pregnancy prediction model were calculated. Sensitivity and specificity points were plotted in the ROC curve, where the *x*-axis denotes “1-specificity,” whereas the *y*-axis represents “sensitivity.” An ROC curve with good predictive performance showed an AUC close to 1.

## Results

### Analytic flowchart for clinical pregnancy prediction

**Fig 1:**
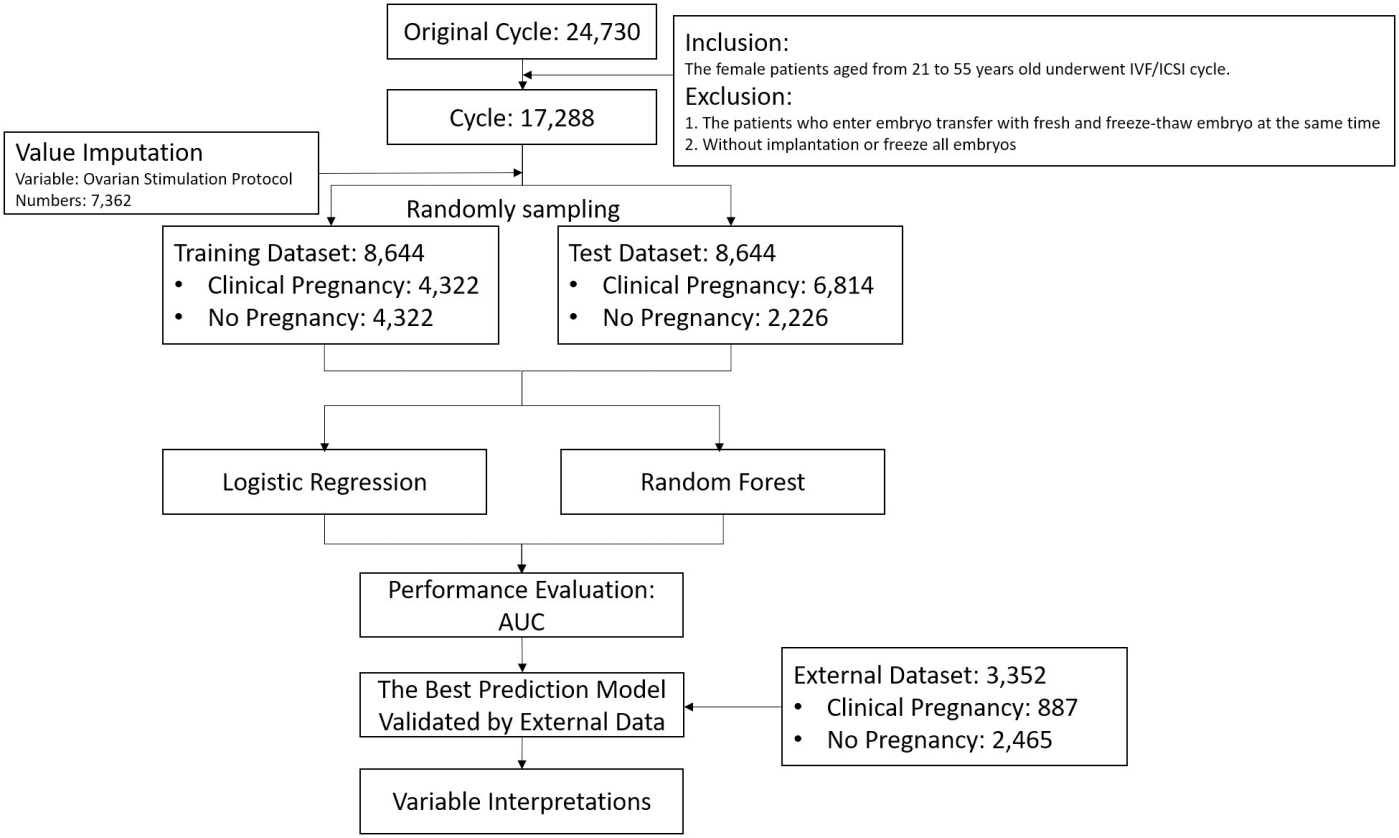
Experiment flowchart for predicting clinical pregnancy outcomes.

The workflow of this study is shown in Fig 1. The original dataset included 24,730 records. We excluded patients who were simultaneously administered fresh and freeze-thawed embryos, those without implantation, and those with frozen embryos. In addition, we randomly sampled patients with the same features to avoid bias in elderly patients to improve prediction accuracy. In total, 7,362 (approximately 42.58%) missing values of the ovarian stimulation protocol were imputed based on the missForest package in the R environment. With the exception of the natural cycle and others, all other missing values were imputed. In addition, the dataset was divided into two parts, and machine learning algorithms were compared based on the predictive performance of test dataset in terms of AUC. Finally, the effect of each analytic variable on clinical pregnancy was depicted.

### Characteristics of the datasets

In total, 17,288 women were analyzed in this study, and 37.88% of women (aged 36.07±3.85 years) achieved a clinical pregnancy. The chi-squared test was used to examine the independence between categorical variables. As shown in Table 2, all variables, except for artificially assisted reproductive methods and sources of sperm and oocytes, showed statistical significance in clinical pregnancy. Student’s *t*-test was used to examine whether the means of continuous variables showed statistically significant differences (Table 3).

**Table 2:** Descriptive statistics of categorical variables for clinical pregnancy and non-clinical pregnancy groups.

*N* = 17,288	Pregnancy (*N* = 6,548)		No pregnancy		*p*-value
			(*N* = 10,740)		
	*N*	Percentage	*N*	Percentage	
Female age group					***
<30 years	294	4.49	267	2.49	
30~34 years	1903	29.06	2091	19.47	
35~39 years	3124	47.71	4425	41.20	
>40 years	1227	18.74	3957	36.84	
Cause of infertility					***
Tubal factor	902	13.78	1335	12.43	
Ovary factor	607	9.27	826	7.69	
Endometriosis	691	10.55	1037	9.66	
Uterine factor (myoma adenomyosis, uterine synechia)	306	4.67	454	4.23	
Others (other female factors)	2078	31.73	3962	36.89	
Male factor	985	15.04	1311	12.21	
Either two factors	940	14.36	1779	16.56	
Unexplained	39	0.60	36	0.34	
Oocyte retrieval					**
Yes	4416	67.44	7412	69.01	
No	2132	32.56	3328	30.99	
Artificially assisted reproductive method					
IVF/ET	6544	99.94	10,739	99.99	
ZIFT/TET	4	0.06	1	0.01	
Fertilization method					***
ICSI	4375	66.81	7352	68.45	
Assisted hatching	223	3.41	226	2.10	
ICSI and assisted hatching	13	0.20	27	0.25	
PGS	121	1.85	117	1.09	
PGD	0	0.00	0	0.00	
No Use	1760	26.88	2943	27.40	
Others	56	0.86	75	0.70	
Source of sperm and oocytes					
Without donation	6445	98.43	10,596	98.66	
Oocyte donation	75	1.15	93	0.87	
Sperm donation	28	0.43	51	0.47	
The use of fresh/freeze-thaw					**
Fresh	4406	67.29	7456	69.42	
Frozen	2142	32.71	3284	30.58	
Ovarian hyperstimulation syndrome					***
None	6323	96.56	10,526	98.01	
Mild	218	3.33	212	1.97	
Moderate	6	0.09	1	0.01	
Severe	1	0.02	1	0.01	
Ovarian stimulation protocol					***
Natural cycle (including frozen-embryo transfer cycle)	2132	55.84	3328	54.51	
Oral stimulation drug	0	0.00	3	0.05	
Short protocol	248	6.50	442	7.24	
Long protocol	306	8.01	356	5.83	
Ultra-long protocol	124	3.25	165	2.70	
Antagonist protocol	1006	26.35	1803	29.53	
Others	2	0.05	8	0.13	

* p < 0.05,

** p < 0.01,

*** p < 0.001 by a chi-squared test for categorical variables.

IVF/ET, in vitro fertilization/embryo transfer; ZIFT/TET, zygote intrafallopian transfer/tubal embryo transfer; ICSI, intracytoplasmic sperm injection; PGS, preimplantation genetic screening; PGD, preimplantation genetic diagnosis.

**Table 3:** Descriptive statistics of continuous variables for clinical pregnancy and non-clinical pregnancy groups.

Variable	Pregnancy	No pregnancy	*p*-value
	(*N* = 6548)	(*N* = 10,740)	
	(M±SD)	(M±SD)	
Duration of infertility (years)	3.73±2.75	4.24±3.04	***
Number of IVF cycles performed	0.99±1.48	1.27±1.77	***
Number of oocytes retrieved	6.96±7.22	5.17±6.02	***
Embryos	5.62±4.11	4.23±3.38	***
The Number of Embryos Transferred	2.66±0.89	2.58±1.01	***
The Total Number of Frozen Embryos	2.79±3.76	1.53±3.00	***
Female age (years)	36.07±3.85	37.94±4.34	***
Male age (years)	38.29±5.02	39.87±5.52	***

Abbreviations: M, mean; SD, standard deviation; IVF, in vitro fertilization

*** p < 0.001 by Student’s t-test for continuous variables.

### Predictive performance of clinical pregnancy

**Fig 2:**
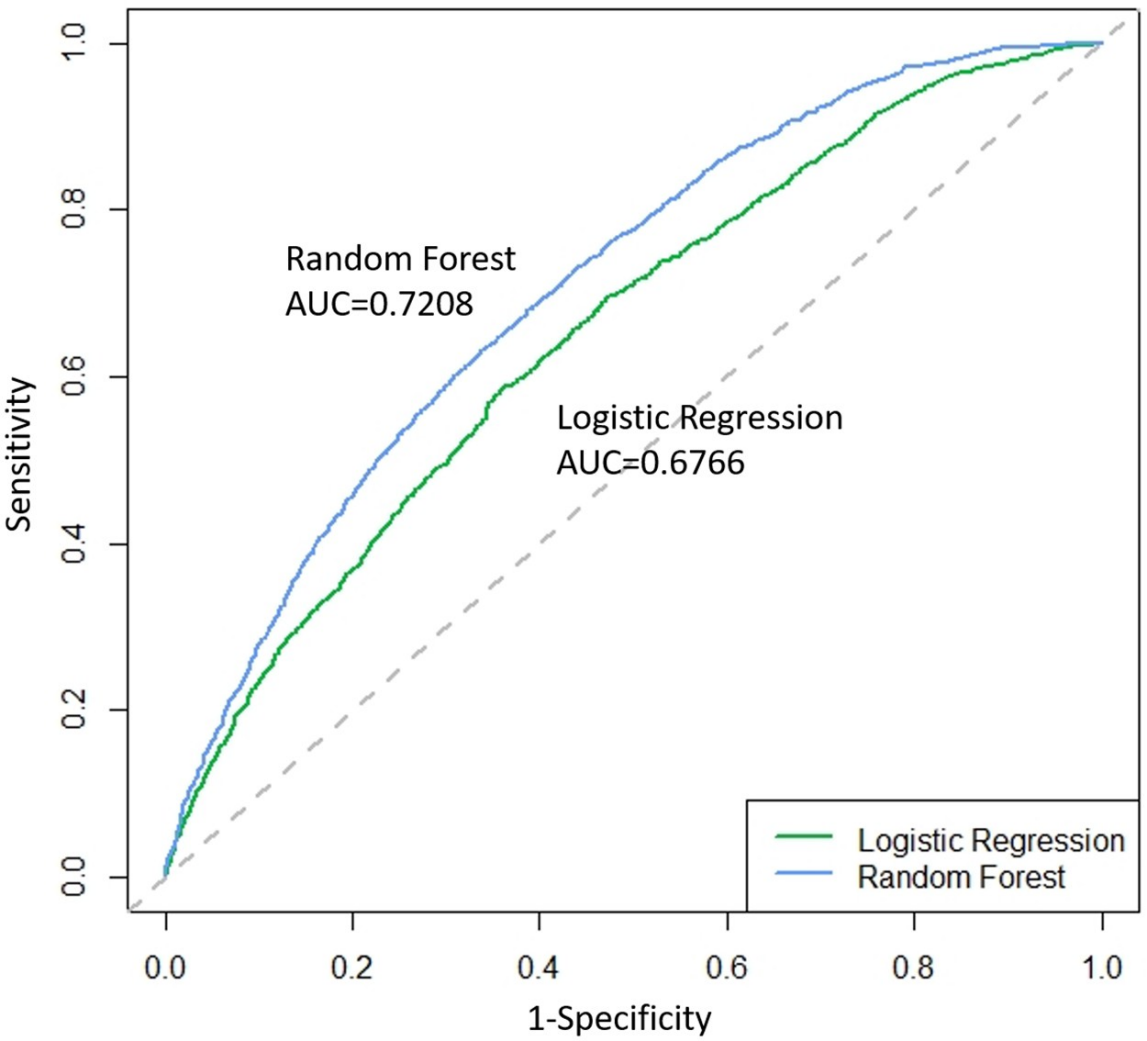
Receiver operating characteristic (ROC) curves and the area under the ROC curve (AUC) for clinical pregnancy predictions based on the logistic regression and random forest for the test set.

The original dataset was randomly separated into training and test datasets. According to the results generated based on the random forest model, the best “mtry” equals 3 with the minimum OOB error and maximum AUC (shown as S1, S2 Figs in S1 File). In the training set, the AUC was used as the main evaluation measure to assess the clinical pregnancy performance of the prediction model. Four measures were used to assess the training dataset (accuracy: 62.20% vs. 83.39%; sensitivity: 62.05% vs. 83.02%; specificity: 62.36% vs. 83.76%; AUC: 0.9197 vs. 0.6783) and the test dataset (accuracy: 62.78% vs. 64.78%; sensitivity: 61.81% vs. 66.58%; specificity: 63.12% vs. 64.16%; and AUC: 0.6766 vs. 0.7208) in the random forest vs. logistic regression prediction models. Table 4 shows that the random forest model outperformed the logistic regression model in terms of accuracy, sensitivity, specificity, and AUC. In Fig 2, the ROC curve of the random forest is closer to the upper left corner of the diagram. Therefore, the random forest model was chosen as the best model for predicting clinical pregnancy outcomes in our study.

**Table 4:** Predictive performance of different machine learning algorithms for the training and test datasets.

Algorithm	Dataset	Number	Accuracy (%)	Sensitivity (%)	Specificity (%)	AUC
Logistic regression	Training	8644	62.20	62.05	62.36	0.6783
	Test	8644	62.78	61.81	63.12	0.6766
Random forest	Training	8644	83.39	83.02	83.76	0.9197
	Test	8644	64.78	66.58	64.16	0.7208

AUC, area under the receiver operating characteristics curve.

### External validation using an independent dataset

To further validate the prediction model, we collected 3,352 new samples from 2019 to 2020 as the external dataset to evaluate the performance of our prediction model. The training and test data sets were combined together to construct a prediction model, and the external data set was used as an independent test set to evaluate the true predictive performance. As shown in Table 5, the predictive performance of the external dataset is similar to the original test dataset (accuracy: 64.78% vs. 62.98%; sensitivity: 66.58% vs. 68.55%; specificity: 64.16% vs. 60.97%; and AUC: 0.7208 vs. 0.7123). This demonstrated that the proposed prediction model did not show overfitting of predictive performance, and thus it can be generalized to predict the clinical pregnancy rate of other patients.

**Table 5:** Predictive performance based on random forest for the training, test, and external dataset.

Dataset	Year	Number	Accuracy (%)	Sensitivity (%)	Specificity (%)	AUC
Training	2007–2018	8644	83.39	83.02	83.76	0.9197
Test		8644	64.78	66.58	64.16	0.7208
External	2019–2020	3352	62.98	68.55	60.97	0.7123

### Variable importance ranking

**Fig 3:**
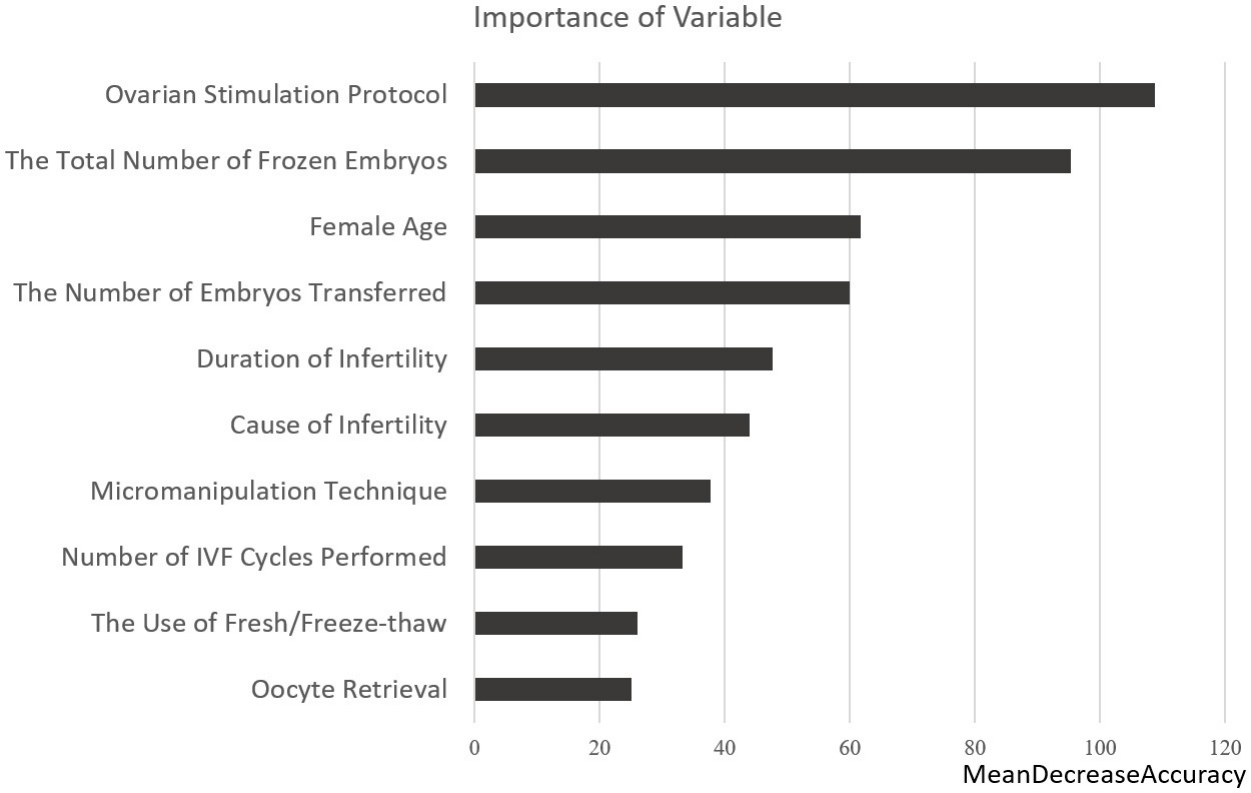
Rank of importance of variables based on random forest clinical pregnancy predictions.

In the random forest model, the function varImpPlot() can generate a partial dependency plot of the importance of variables by measuring the mean decrease in accuracy (MDA). The results showed that the ovarian stimulation protocol, the total number of frozen embryos, and female age were the most important variables in the analysis. We discuss how variables affect the probability of clinical pregnancy. Important variables affecting clinical pregnancy outcomes were further analyzed to determine the relationships between predictors and clinical pregnancy. These results can help us understand the relationship between the marginal effects of predictors and clinical pregnancy (Fig 3).

### Correlations between clinical variables and pregnancy

**Fig 4:**
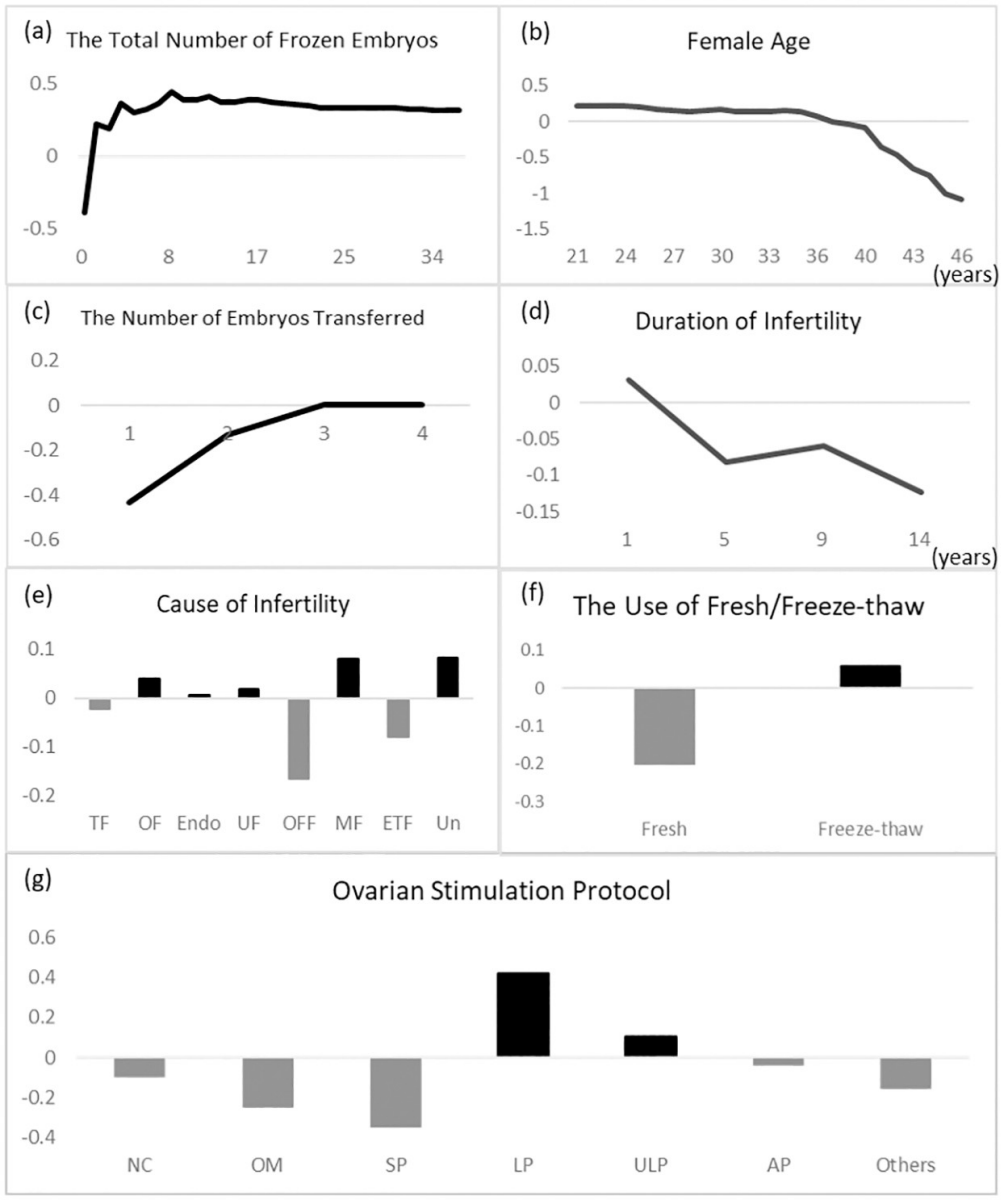
Partial dependence plots of the most influential continuous and categorical variables for clinical pregnancy outcomes analyzed for (a) the total number of frozen embryos, (b) female age, (c) the number of embryos transferred, (d) duration of infertility, (e) cause of infertility, (f) the use of fresh/freeze-thaw, and (g) ovarian stimulation protocol. Continuous variables are presented as line plots, and categorical are presented as bar plots.

The correlations between analytic variables and clinical pregnancy depicted by partial dependency plots were shown in Fig 4. The total number of frozen and transferred embryos was positively correlated with clinical pregnancy outcomes, whereas the female age and duration of infertility were negatively correlated. The total number of frozen embryos showed a non-linear relationship with clinical pregnancy outcomes. As shown in Fig 4a, the marginal effect positively increased in eight frozen embryos and began to gradually decrease. The female age showed an obvious negative effect. Women < 36 years old exhibited constant conditions. After 36 years of age, there was a negative propensity for clinical pregnancy. After 40 years of age, the probability of a clinical pregnancy dramatically declined (Fig 4b). As shown in Fig 4c, the propensity of clinical pregnancy was positively correlated with the number of embryos transferred in the same IVF cycle, and the transfer of three and four embryos had little effect on clinical pregnancy compared to one or two, which showed a greater propensity of becoming pregnant. The duration of infertility was negatively correlated with clinical pregnancy. After 1 year of infertility, the propensity decreased dramatically (Fig 4d). Compared to other causes of infertility, other female factors and either of the two factors were the main factors that decreased the probability of a clinical pregnancy (Fig 4e). The use of frozen embryos was positively correlated with clinical pregnancy, while the transfer of fresh embryo was negatively correlated (Fig 4f). Long and ultra-long protocols were two effective treatments compared to the other protocols (Fig 4g).

## Discussion

### Significance and contribution of this study

This study attempted to identify variables that could affect clinical pregnancy outcomes and interactions among different variables. Twelve variables were used for analysis in this study. Data were collected from IVF patients between 2007 and 2019 for the model development. Our results demonstrated that 37.88% of women achieved a clinical pregnancy. After developing our predictive model, the random forest model outperformed the logistic regression model for all measures. Among all variables, we found that the “ovarian stimulation protocol” was the most important variable, and long and ultra-long protocols had positive effects on achieving a clinical pregnancy. The second most important variable was the total number of frozen embryos, which had positive effects, peaked at eight, and then began to flatten out.

Data of patients from TMUH were connected to a database of the Health Promotion Administration, Ministry of Health and Welfare, Taiwan. The dataset contained >15,000 IVF cycles with 50 variables. Among these, 42.6% of the “ovarian stimulation protocol” variables in our dataset contained missing values. We compared three functions to impute the missing values. Finally, we incorporated the dataset imputed by the “missForest” function for the analysis.

Another strength of our study is that we provide the importance and propensity of each variable to achieve a clinical pregnancy. Most studies used logistic regression to construct prediction models for pregnancy and live births [19–22], and ORs were used to explain the marginal effects of each variable. Our model comparison results showed that the random forest model outperformed the logistic regression model in terms of accuracy and AUC. In addition, the effects of each continuous variable on the clinical pregnancy probability were nonlinear, and partial dependency plots could be used to illustrate positive or negative effects on clinical pregnancy outcomes. Previous studies have demonstrated that the day of transfer is an important variable associated with pregnancy [19, 23]. In our study, the number of embryos transferred did not distinguish between the day of transfer.

The AUC evaluates the performance of the prediction model. The AUC of the test dataset was 0.7208, and was comparable with other previous studies based on logistic regression with AUCs ranging 0.6–0.7 [7–9]. Our study showed that the random forest model is a good algorithm for classifying clinical pregnancies. Kaufmann *et al*. reported an accuracy of 58.8% for predicting outcomes of IVF using neural networks [24], and Blank *et al*. reported that the average AUC, sensitivity, and specificity were 0.74, 0.84, and 0.48, respectively [11].

### Clinical interpretations of analytic variables

#### (1) Female age

The baseline characteristics of the female age group were significantly correlated with clinical pregnancy outcomes as shown in Table 2 and Fig 2b, and the mean female age in the clinical pregnancy group was younger than that in the group without clinical pregnancy. In the random forest model, the female age was a non-linear curve and showed negative effects on clinical pregnancy outcomes. The probability of a clinical pregnancy dramatically declined after the age of 40 years. Compared to other studies, it is a common predictor in prediction models of clinical pregnancy or live births and has negative effects on clinical pregnancy outcomes [8, 19, 21, 25, 26]. This can be explained by aneuploidy in embryos increasing with maternal age [27], which weakens their developmental potential [28].

#### (2) Freeze-thawed embryos or fresh embryos

As shown in Fig 4f, there were more freeze-thawed embryos than fresh embryos. Table 2 shows the rate of pregnancy with freeze-thawed embryos that was 2% higher than that with fresh embryos. In recent studies, the pregnancy rate in frozen embryos was higher than that in fresh embryos [29–32]. Freeze-thawed embryo technology plays a key role in IVF because traditional freezing technology has greatly improved [33]. The number of transfer cycles of freeze-thawed embryos has increased over the past decade, and the technology of freeze-thawed embryo transfer enhanced the live birth rate [34]. Chen *et al*. found that the advantage of frozen-embryo transfer was correlated with a higher live birth rate and lower ovarian hyperstimulation syndrome risk for infertile women with polycystic ovary syndrome [35]. They also observed that frozen embryo transfer allows the ovary to recover from ovarian stimulation and the estradiol levels. Although there is evidence that freeze-thaw embryo transfer has a significant effect on live births, its effect on clinical pregnancies is uncertain. Wang *et al*. reported that the rate of clinical pregnancy with freeze-thaw embryo transfer was higher than that with fresh transfer, but the difference was not significant [33]. Our results showed that the freeze-thaw transfer group was more likely to achieve pregnancy than the fresh group. Previous studies reported that the clinical pregnancy rate of the cryopreservation group was significantly higher than that of the fresh group [36, 37]. Shapiro *et al*. reported that the pregnancy rate with freeze-thawed embryo transfer cycles was greater than that with fresh cycles may be because the typical freeze-thawed embryo transfer cycle uses suboptimal embryos for cryopreservation after their superior siblings are transferred in a fresh state. To keep endometrium steady without ovarian hyperstimulation syndrome (OHSS), intentionally implanting freeze-thaw embryos but not fresh ones to patients in the clinical practice is conducted when the total number of egg retrieval is greater than 20 and estradiol (e2) is greater than 3000. In addition, many freeze-thaw protocols are inadequate to confirm recovered embryo development [37]; therefore, fresh or frozen embryo transfer remains controversial.

#### (3) Cause of infertility

Our results showed that the ovary factor, the male factor, and unexplained reasons were more likely to achieve pregnancy through IVF compared to other factors in Fig 4e. In previous studies [21, 38, 39], infertility was found to be the main predictor of clinical pregnancy outcomes. The male factor, ovary factor, and unexplained reasons were obviously more important in clinical pregnancy outcomes than tubal factors, which is in line with a previous study [21]. Infertile women due to male factors can be successfully impregnated with IVF. Other female factors, including recurrent miscarriages and higher prolactin levels, are harmful to clinical pregnancy outcomes. Any of the factors raise the difficulty of becoming pregnant due to interactions. In previous studies, women with unexplained infertility had a higher live birth rate per embryo transfer than those with other causes of infertility [39].

#### (4) Ovarian stimulation protocols

Ultralong protocol and long protocol has have been one of the standard protocols for ovarian hyper stimulation. Using Gonadotropin-releasing hormone (GnRH) antagonist for ultralong protocol can tackle cases with endometriosis. GnRH agonist is able to lower then effects of endometriosis, in the meanwhile we are able to perform embryo transfer in the same cycle. In short protocol, the stimulation period is relatively short without pretreatment. Therefore, we believe that the negative effect on short protocol compare to long or ultra-long protocol is without pretreatment with GnRH agonist.

#### (5) The numbers of embryos transferred

Higher embryo transfer numbers increase the probability of achieving a clinical pregnancy as shown in Fig 4c. The transfer of more embryos results in more clinical pregnancies. Baker *et al*. used logistic regression to analyze data and found that a higher number of embryos transferred increased the probability of achieving pregnancy [20].

#### (6) Duration of infertility

In our study as shown in Fig 4d, infertile women with more than 1 year of infertility were less likely to become pregnant than those with less than 1 year of infertility, and the duration showed negative effects on clinical pregnancy outcomes. IVF increases the probability of pregnancy and has the best effect on infertility in the first year. A longer duration of infertility is correlated with a reduction in the possibility of conception, but the effects on IVF outcomes remain unclear. Templeton *et al*. reported a significant reduction in the success rate of IVF due to infertility with a longer duration of infertility [40]. Several studies have reported that the duration of infertility is a predictor of clinical pregnancy. Similar to most studies, the duration of infertility was treated as a continuous variable in our analysis. Ottosen *et al*. used the duration as a categorical variable to analyze the effects on a pregnancy model. Their results showed that the duration of infertility had a negative nonlinear effect [8]. Compared with the consensus of the duration of infertility in other studies, the probability of clinical pregnancy decreases after the first year [8, 9, 19].

### Highlights and limitations of the proposed study

Our findings highlight the importance of certain variables and the propensity of each variable by machine learning with the random forest model to result in a clinical pregnancy in ART cycles. In the future, the model can also be used to predict the probability of a clinical pregnancy for individual patients, suggestive of methods to assist a patient in becoming pregnant. We will include embryo image characteristics, hormone profiles, and embryo grading along with this dataset to improve prediction ability. In the future, images and grading of embryos can be combined with the important variables suggested in this study to achieve better predictions for achieving a clinical pregnancy. The limitations of our study include data integrity, and some variables used in previous studies were lacking for further discussion, such as BMI, embryo grading, details of the stimulation protocol except for the drug dose, timing, AMH, basic hormone, and semen analysis. Incorporated variables can influence the ability to predict clinical pregnancy. Furthermore, information on lifestyle-related factors and disease status is limited.

## Conclusions

Our analysis showed that RF outperformed logistic regression for predicting clinical pregnancy outcomes. In our results, the AUC values of the test dataset with the logistic regression and random forest models were 0.6766 and 0.7208, respectively. The performance of logistic regression was similar to those of previous studies, and the random forest model outperformed them all.

## Supporting information

S1 File (DOCX)
