# Supplementary material for: Predicting clinical pregnancy using clinical features and machine learning algorithms in in vitro fertilization
Source: PLoS One. 2022 Jun 8;17(6):e0267554. doi: 10.1371/journal.pone.0267554 (PMC9176781; doi:10.1371/journal.pone.0267554)
Supplement: S1 File — (DOCX) [file pone.0267554.s001.docx]

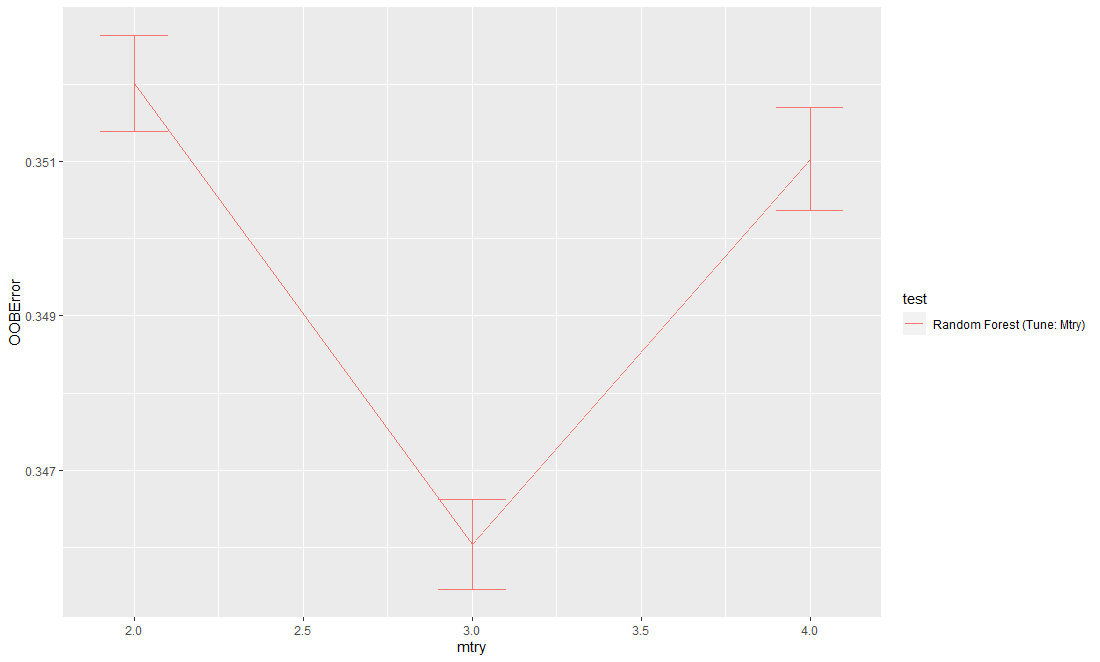


**Figure S1 Hyperparameter tuning of the *mtry* parameter in the random forest model based on the minimum out-of-bag error for 10 times.**


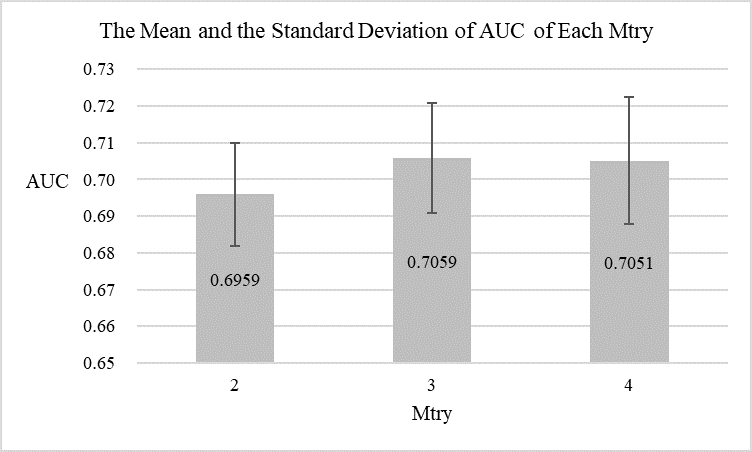


**Figure S2 Hyperparameter tuning of the *mtry* parameter in the random forest model based on the maximum AUC in ten-fold cross-validation.**
